# Attosecond-Level Delay Sensing via Temporal Quantum Erasing

**DOI:** 10.3390/s23187758

**Published:** 2023-09-08

**Authors:** Fabrizio Sgobba, Andrea Andrisani, Stefano Dello Russo, Mario Siciliani de Cumis, Luigi Santamaria Amato

**Affiliations:** 1 Italian Space Agency (ASI), Centro Spaziale ‘Giuseppe Colombo’, Località Terlecchia, 75100 Matera, Italy; fabrizio.sgobba@asi.it (F.S.); andrea.andrisani@asi.it (A.A.); stefano.dellorusso@asi.it (S.D.R.); mario.sicilianidecumis@asi.it (M.S.d.C.); 2 Istituto Nazionale di Ottica, Consiglio Nazionale delle Ricerche, L.go E. Fermi 6, 50125 Firenze, Italy; 3 Istituto Nazionale di Ottica, Consiglio Nazionale delle Ricerche, Via Campi Flegrei N. 34, 80078 Pozzuoli, Italy

**Keywords:** Hong-Ou-Mandel interferometry, polarization entanglement, quantum eraser

## Abstract

Traditional Hong-Ou-Mandel (HOM) interferometry, insensitive to photons phase mismatch, proved to be a rugged single-photon interferometric technique. By introducing a post-beam splitter polarization-dependent delay, it is possible to recover phase-sensitive fringes, obtaining a temporal quantum eraser that maintains the ruggedness of the original HOM with enhanced sensitivity. This setup shows promising applications in biological sensing and optical metrology, where high sensitivity requirements are coupled with the necessity to keep light intensity as low as possible to avoid power-induced degradation. In this paper, we developed a highly sensitive single photon birefringence-induced delay sensor operating in the telecom range (1550 nm). By using a temporal quantum eraser based on common path Hongr-Ou-Mandel Interferometry, we were able to achieve a sensitivity of 4 as for an integration time of $2\cdot 10^{4}$ s.

## 1. Introduction

Since its discovery, Hong-Ou-Mandel (HOM) interference [1] as proved to be a resourceful phenomenon for a plethora of applications in the emerging field of Quantum Technology (see [2] for a comprehensive review).

Namely, when two perfectly indistinguishable photons impinge on two input ports of a beam splitter (BS), they “bounce” together as a consequence of their bosonic nature, coming out from the same output port of the BS.

In sensing applications, HOM interference proved its broad applicability from quantum state tailoring [3,4,5] to single photon interferometry [6,7], where the HOM effect is especially suited to reach Heisenberg limit in quantum metrology [8,9], since it is employed to generate quantum optical NOON states.

Developing a sensor based on HOM interferometry offers several advantages over classical interferometry.The typical HOM interferometry setup is by its own nature more robust and far easier to implement since it does not require complex schemes for interferometer stabilization, as the HOM effect is independent of the relative phase between the two interfering photons.

HOM interferometry lacks invasiveness, since it operates at the single photon level, and therefore it is particularly suitable for applications to biological samples or optical metrology, two fields where even a low light intensity can induce respectively unwanted chemical reactions or accuracy degradation.

Moreover, unlike classical interferometry, HOM interferometry is not subjected to the half wavelength ambiguity range, caused by the periodicity of the acquired signal.

Over time, thanks to technology advancements [10,11] in single photon detection [12,13], light engineering [14,15,16], and innovative schemes [17,18,19,20,21], HOM-based interferometry has seen a constant improvement in its performance, broadening even further the horizons of its applications.

The HOM effect cannot be pictured merely as a single-photon counterpart to the classical light interference, since, as shown in [22,23], the dip in coincidence events can be retrieved even if a couple of distinguishable photons impinging the BS are adopted, provided that such distinguishability is erased after the BS and before the detection event [24] (similarly to the “spatial” quantum eraser described in [25,26], that is based on a Young interferometer).

Taking advantage of this effect, Dauler et al. [27] developed a common path HOM interferometer by recovering the indistinguishability through two polarizers placed after the beamsplitter, and measured the polarization mode dispersion of birefringent media with a resolution of 200 as. One year later Branning et al. [28] measured the group and phase delay experienced by two orthogonally polarized photons traveling through a birefringent crystal with an uncertainty of 100 as and 8 as, respectively.

In 2018, Lyons et al. [29] introduced a quantum information-based model by which they achieved a precision of a few attoseconds in a HOM interference experiment with non-copropagating photons, therefore applicable to non-birefringent samples as well. This result was obtained by performing long-term measurements around the time delay (δ) where the Fisher information function F(δ) assumes its maximum value. It is worth to recall that, according to [30], “for a parameter δ and measurement outcomes m∈M with P(m|δ) the probability of outcome *m* given δ, the Fisher information can be written“

$$F\left(\delta \right)=\sum_{m\in M}\frac{1}{P(m|\delta )}{\left(\frac{\partial }{\partial \delta }P\left(m|\delta \right)\right)}^{2}$$

In this paper, the pioneering experiment proposed by [29] in the optical frequencies range is reinterpreted with a common path HOM interferometer, obtaining sensitivities of a few attoseconds (comparable with the state of the art) but, for the first time in the literature, in the telecom wavelength range, therefore opening the path for the adoption of this metrologic technique to fiber-coupled devices up to hundreds of kilometers long as optical networks and gyroscopes.

## 2. Experimental Setup

**Figure 1:**
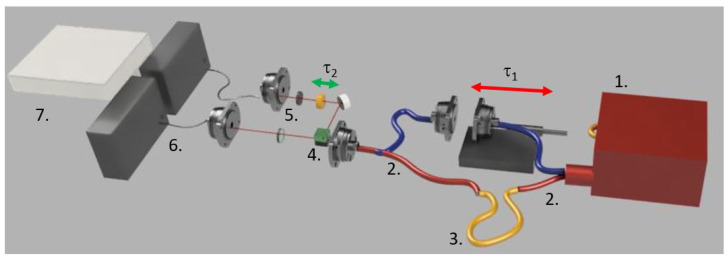
Representation of the setup for delayed-choice temporal quantum eraser: (1.) spontaneous parametric down conversion twin photon source; (2.) fiber-coupled polarising beam splitter/combiner; (3.) polarization-maintaining fibre patch cable; (4.) balanced beam splitter; (5.) linear absorptive polarizers mounted on motorized rotational stages; (6.) InGaAs single photon avalanche diodes detectors; (7.) time tagging device or coincidence recording. Adjustable time delays imparted to photons during the experiment are represented by red (translation stage) and green (voltage-driven tunable waveplate) arrows This Figure has been drawn in Fusion 360.

The proposed sensor employs a Twin Photons Source (TPS, 1. in Figure 1) equipped with a Continuous Wave (CW) laser centered at 775 nm, pumping a Periodically Poled Lithium-Niobate (PPLN) crystal designed to attain the best performance in terms of type-II Spontaneous Parametric Down Conversion (SPDC) efficiency at the center downconverted wavelength of 1550 nm and at the actively stabilized temperature of 33.9 °C.

Every photon pair produced inside the TPS is then separated via a fibre-coupled polarizing beam splitter (2. in Figure 1). Horizontally polarized photons propagate through a polarization maintaining connector, whereas vertically polarized photons undergo a tunable delay ($\tau_{1}$ in Figure 1 indicates the temporal mismatch between the two polarizations before the balanced BS) by means of a translation stage. Both photons are coupled back on the same longitudinal mode by means of a second identical polarizing beam splitter, acting in this case as a combiner.

The heralded photon pair then impinges on a balanced BS (BS, 4. in Figure 1), where it separates into two longitudinal modes. Along one of the emerging paths from the BS is placed a tunable waveplate, consisting of a voltage-driven liquid crystal optimized with anti-reflection coating to operate in the telecom spectral region (Thorlabs LCC1115-C). By means of this crystal, it is possible to apply a tunable time delay ($\tau_{2}$) between photons with different polarizations. Finally, photon pairs impinge on two absorptive polarizers that select the polarization (5. in Figure 1), one for each longitudinal mode, before interacting with two thermo-electrically cooled Single Photon Avalanche Diode (SPAD, 6. in Figure 1) detectors, placed just behind the polarizers. The two SPADs are connected by a time-tagging device (7. in Figure 1), in order to record both single counts on each detector and coincidence counts between detectors as well.

## 3. Results and Discussion

### 3.1. Quantum Eraser Interferogram

If the polarisers are set in the lDD-configuration, that is, positioned with the respective transmission axis at angles $\left\{\frac{\pi }{4},\frac{\pi }{4}\right\}$ with respect to the horizontal axis of the final output fibre-port, it is possible to retrieve the characteristic Hong-Ou-Mandel dip, given by the so-called “photon bouncing”, by adjusting the delay $\tau_{1}$ imparted before the BS [27,28]. In the proposed setup, it is possible to modify $\tau_{1}$ via the translation stage (red arrow in Figure 1) achieving the condition of minimum distinguishability, characterized by a minimum in recorded coincidence events. The condition ($\tau_{1}=0$) results are critical to obtaining the best possible sensor performance.

**Figure 2:**
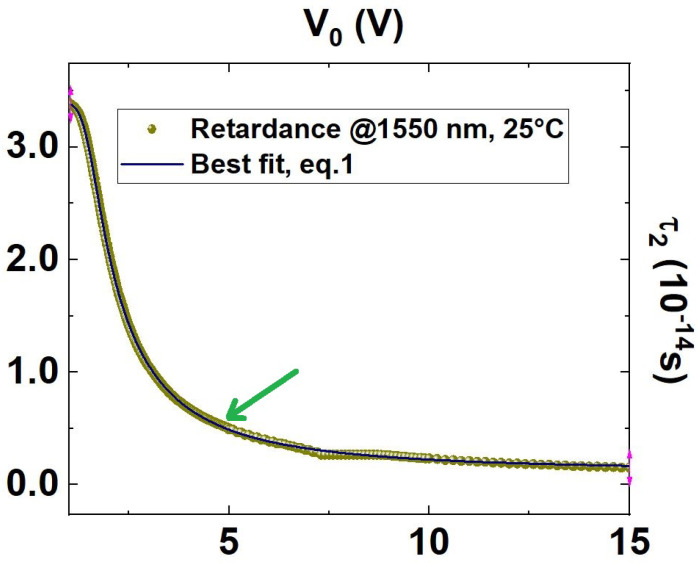
Delay of vertically polarized mode with respect to the horizontally polarized mode as a function of the applied voltage $V_{0}$ (dark yellow dots), for radiation at 1550 nm, and crystal temperature of 25 °C; data retrieved by converting the retardance reported in Thorlabs datasheet. The data points are fitted with the logistic function reported in Equation (1) (straight blue line) for calibration. The green arrow shows the point of operating conditions (see $\tau_{2}$ in Table 1).

Once the center of the dip has been reached, the second step consists of adding the tuneable waveplate alongside one of the two output modes of the BS (as shown in Figure 1); note that the electronic delay on the corresponding detector must be accordingly modified to even out the additional path. If the optical axis of the tuneable waveplate is aligned with the horizontal axis of the output fibre-port, by varying the applied voltage ($V_{0}$) to the tuneable waveplate it is possible to introduce a controllable polarization-dependent time delay (see Figure 2), that results in a polarization-dependent variable phase mismatch between the entangled photons.

It is possible to fit the expected imparted delays $\tau_{2}$ as a function of the applied voltage to calibrate the waveplate behavior. The best fit, used as a calibration function, results to be

(1) $$\tau_{2}\left(V_{0}\right)=1.18\cdot 10^{-15}\;s+\frac{\left(3.40\cdot 10^{-14}\;s-1.18\cdot 10^{-15}\;s\right)}{{\left(1+{\left(\frac{1.50\;V}{V_{0}}\right)}^{-9.18}\right)}^{0.2}}.$$

It is worth mentioning that if the logistical behavior of data reported in Figure 2 and reproduced by Equation (1) could be intuitively explained by the working principle of the tunable waveplate, where the applied voltage acts on the pre-existing alignment of the polymers within the crystal progressively reducing the delay experienced between orthogonal polarizations, the introduced parameters are only meant to obtain the best fitting function (calibration curve) and do not have direct physical meaning.

If a delay before the beam splitter ($\tau_{1}$) allows appreciating the carrier dip of the HOM interference pattern (in the tens or hundreds of fs scale), with fine-tuning of the polarization-dependent delay $\tau_{2}$, by contrast, it is possible to appreciate the fringes within the dip, allowing unlocking sensitivities in the phase delays measurements below the fs scale [27,28].

The twin photon pair is detected by measuring the occurrence of coincidence events within a certain coincidence window. In order to rule out fluctuations of detectors and coupling efficiency the figure of merit *R* is introduced

(2) $$\begin{array}{c} R=\frac{N_{ab}}{\sqrt{N_{a}N_{b}}}, \end{array}$$

representing the measured coincidence events ($N_{ab}$) at a certain integration time *T* normalised over the total amount of detectable coincidences ($\sqrt{N_{a}N_{b}}$, being $N_{a/b}$ the detected single count events during the time *T*). Note that *R* does not depend on *T*.

**Figure 3:**
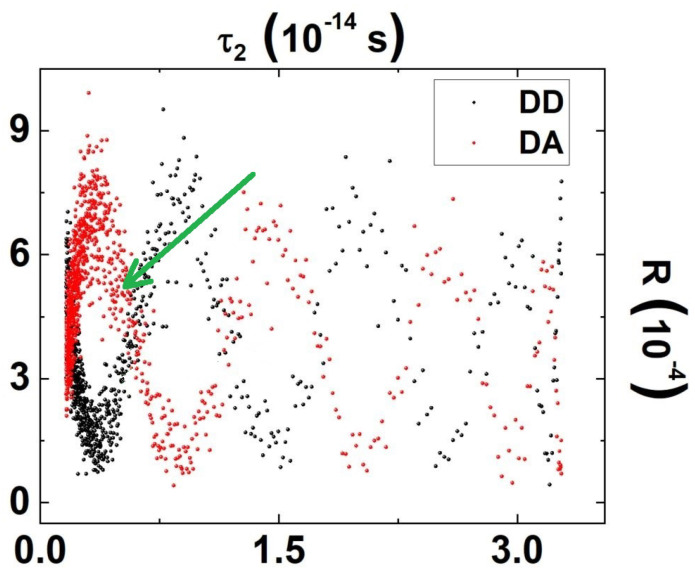
DD-configuration (black dots) and DA-configuration (red dots) interferogram as a function of the applied delay $\tau_{2}$. The minimum in *R* is referred to as “photon bouncing”, whereas the maximum is “anti-bouncing”. The green arrow shows the operative conditions for $\tau_{2}$, see Table 1.

A LabVieW-based script able to assign 1000 equally spaced voltage steps (between $V_{0}=1$ V and $V_{0}=15$ V) to the tunable waveplate and to measure coincidence and single count events on both detectors has been employed to retrieve the HOM interference pattern reported in Figure 3, both in DD and DA = $\left\{\frac{\pi }{4},\frac{3\pi }{4}\right\}$ configurations. To perform these measurements, we employ an integration time T=5 s (plus an additional time $T^{\prime }=1$ s for tunable waveplate adjustment between different voltages), and a coincidence window $\tau_{W}=250$ ps.

With reference to Figure 3, the visibility 𝒱 of the first measurable fringe results

$$𝒱=\frac{R_{DA,max}-R_{DD,min}}{R_{DA,max}+R_{DD,min}}>65\%.$$

### 3.2. Choice of Operating Conditions

In Figure 2, it can be observed how evenly spaced steps in voltage generate unevenly spaced delays $\tau_{2}$ due to the nonlinear dependence typical of the tunable waveplate.

As the operative condition for the proposed delay sensor has been chosen, the applied voltage $V_{0s}=4.70$ V, corresponding to a delay $\tau_{2s}∼5.1fs=0.51\times 10^{-14}$ s, which results in being the point in the best responsivity region of the tunable waveplate, that is nearest to the inflection point of the first fringe of the interferogram $R(\tau_{2})$ (compare with Figure 3).

All the working conditions of the proposed delay sensor are summarized in Table 1.

### 3.3. Sensor Stability and Detection Limit

A long-term (>11 h) measurement has been devised and performed in the operating conditions reported in Table 1, simulating the introduction of the birefringent sample by slightly changing the applied voltage around $V_{0s}$ ($\Delta V_{0}=0.02$ V, corresponding to an expected $\Delta \tau_{2}∼32$ as).

The delays $\tau_{2}\;\left(4.69\;V\right)$ and $\tau_{2}\;\left(4.71\;V\right)$ have been measured repeatedly by alternating the two voltage values. Since the introduced delay $\Delta \tau_{2}$ resulting from $\Delta V_{0}=0.02$ V can be considered small with respect to the periodicity of the interferometric fringes (Figure 3), and the working condition employed during the measurement, $\tau_{2s}\;\left(V_{0s}\right)$, is close to the the inflection point of $R\;(\tau_{2})$, R measurements linearly map $\tau_{2}$ measurements.

**Figure 4:**
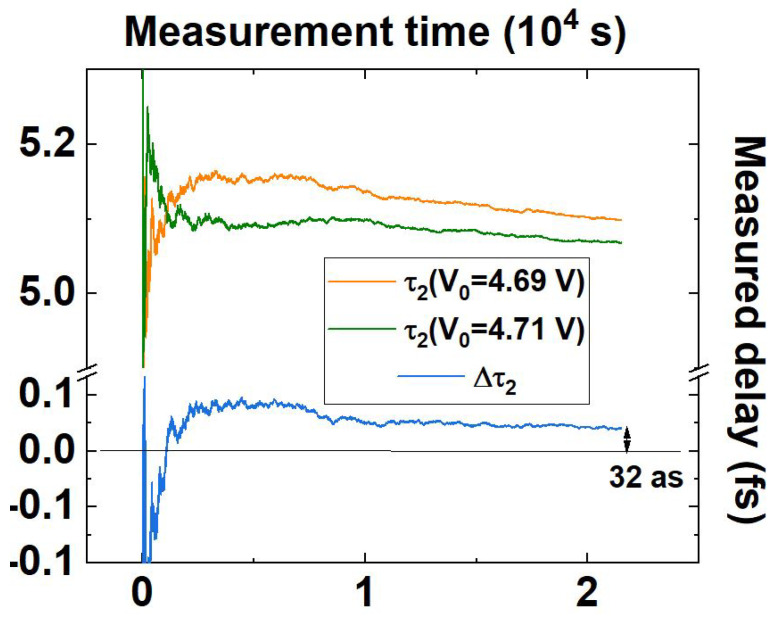
Integral average of the >11 h long-term acquisition, performed alternating an operating voltage of 4.69 V (orange straight line) and 4.71 V (olive straight line). Their difference $\Delta \tau_{2}$ is reported as a blue straight line. The black guideline represents a no-delay condition.

The integral average $\tau_{2ia}$ of the acquired delays, defined as [31]

(3) $$\begin{array}{c} \tau_{2ia}\left(t_{N}\right)=\left(\frac{1}{N}\right)\sum_{i=0}^{N-1}\tau_{2}\left(t_{i}=\left(i+1\right)t_{0}\right), \end{array}$$

is reported in Figure 4 as a function of the averaging time $t_{N}$ ($t_{N}=Nt_{0}$). Here, the acquisition time $t_{0}=2$ s takes into account for the (negligible) period of adjustment of the crystal and the integration time needed to acquire the two values of $\tau_{2}$.

**Figure 5:**
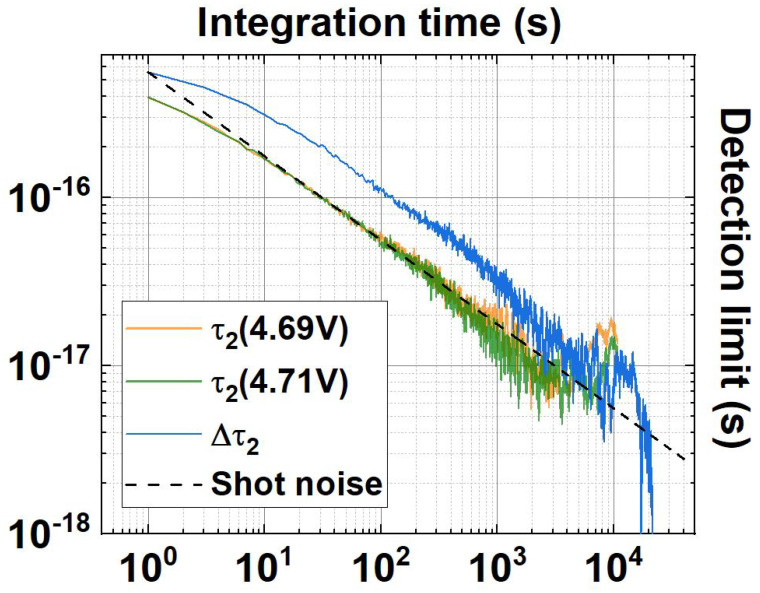
Allan deviation analysis of the >11 h long-term acquisition. The shot noise $\sigma_{0\Delta \tau_{2}}/\sqrt{2t_{N}^{\prime }}$ (black dashed line) is also reported for comparison. The Allan deviations are expressed as detection limits supposing a unitary SNR.

Figure 4 proves that the introduction of a birefringent sample imparting ∼32 as delay within the entangled photon pair can be reliably detected for $t_{N}>1$ h. In order to accurately evaluate the detection limit, an Allan deviation analysis has been performed over the same long-term acquisition. If each individual $\tau_{2}$ has been measured with integration time $t_{0}^{\prime }=1$ s, the delay $\Delta \tau_{2}$, obtained by subtracting the two delays at different operating voltages, has to be intended as measured at $2t_{0}^{\prime }$. The results are reported in Figure 5 as a function of $t_{N}^{\prime }=Nt_{0}^{\prime }$ for $\tau_{2}$ and $2t_{N}^{\prime }=2Nt_{0}^{\prime }$ for $\Delta \tau_{2}$, $N\in [2,N_{tot}/2]$, with ($N_{tot}∼$ 21,000) the total number of datapoints acquired per single $\tau_{2}$.

Both Figure 4 and Figure 5 show how the difference $\Delta \tau_{2}$ is insensitive to the long-term drift affecting $\tau_{2}$ at both operating voltages. At integration time $t=2\cdot 10^{4}$ s the detection limit $\Delta \tau_{2DL}$ for $\Delta \tau_{2}$ results

$$\Delta \tau_{2DL}\approx 3.8\;\mathrm{as}.$$

## 4. Conclusions

In this paper, a temporal quantum eraser based on delayed-choice common-path HOM interferometry has been implemented in the telecom region, retrieving sensitivities as low as 3.8 as, which is fully competitive with past and present literature [27,28,29,31], but represents a record-breaking result for HOM interferometry in the telecom wavelength region (1550 nm) and with partly fibre-coupled setup. The sensor proposed, moreover, is by far more suitable to compaction and susceptible to a wide variety of improvements, ranging from better detection systems (for example superconducting nanowires) to more stable totally fiber-coupled configurations. As such, it paves the way for an extremely promising delay-sensing single photon technique, interesting for optical metrology as well as biological sensing applications. 

## Figures and Tables

**Table 1:** Working conditions chosen for the delay sensor.

SPDC Crystal Temp.	$V_{0s}$	$\tau_{2s}$	$\tau_{1s}$	Polarizers Config.
33.9 °C	4.70 V	5.1 fs	0 fs	DA = $\left\{\frac{\pi }{4},\frac{3\pi }{4}\right\}$

## Data Availability

The data that support the findings of this study are available from the corresponding author L.S.A. upon reasonable request.
